# Microdialysis Assessment of Cerebral Perfusion during Cardiac Arrest, Extracorporeal Life Support and Cardiopulmonary Resuscitation in Rats – A Pilot Trial

**DOI:** 10.1371/journal.pone.0155303

**Published:** 2016-05-13

**Authors:** Andreas Schober, Alexandra M. Warenits, Christoph Testori, Wolfgang Weihs, Arthur Hosmann, Sandra Högler, Fritz Sterz, Andreas Janata, Thomas Scherer, Ingrid A. M. Magnet, Florian Ettl, Anton N. Laggner, Harald Herkner, Markus Zeitlinger

**Affiliations:** 1 Department of Emergency Medicine, Medical University of Vienna, Währingergürtel 18–20, 1090 Vienna, Austria; 2 Department of Neurosurgery, Medical University of Vienna, Währingergürtel 18–20, 1090 Vienna, Austria; 3 Department of Pathobiology, University of Veterinary Medicine of Vienna, Veterinärplatz 1, 1210 Vienna, Austria; 4 Department of Internal Medicine III, Medical University of Vienna, Währingergürtel 18–20, 1090 Vienna, Austria; 5 Department of Clinical Pharmacology, Medical University of Vienna, Währingergürtel 18–20, 1090 Vienna, Austria; Scuola Superiore Sant'Anna, ITALY

## Abstract

Cerebral metabolic alterations during cardiac arrest, cardiopulmonary resuscitation (CPR) and extracorporeal cardiopulmonary life support (ECLS) are poorly explored. Markers are needed for a more personalized resuscitation and post—resuscitation care. Aim of this study was to investigate early metabolic changes in the hippocampal CA1 region during ventricular fibrillation cardiac arrest (VF-CA) and ECLS versus conventional CPR. Male Sprague-Dawley rats (350g) underwent 8min untreated VF-CA followed by ECLS (n = 8; bloodflow 100ml/kg), mechanical CPR (n = 18; 200/min) until return of spontaneous circulation (ROSC). Shams (n = 2) were included. Glucose, glutamate and lactate/pyruvate ratio were compared between treatment groups and animals with and without ROSC. Ten animals (39%) achieved ROSC (ECLS 5/8 vs. CPR 5/18; OR 4,3;CI:0.7–25;p = 0.189). During VF-CA central nervous glucose decreased (0.32±0.1mmol/l to 0.04±0.01mmol/l; p<0.001) and showed a significant rise (0.53±0.1;p<0.001) after resuscitation. Lactate/pyruvate (L/P) ratio showed a 5fold increase (31 to 164; p<0.001; maximum 8min post ROSC). Glutamate showed a 3.5-fold increase to (2.06±1.5 to 7.12±5.1μmol/L; p<0.001) after CA. All parameters normalized after ROSC with no significant differences between ECLS and CPR. Metabolic changes during ischemia and resuscitation can be displayed by cerebral microdialysis in our VF-CA CPR and ECLS rat model. We found similar microdialysate concentrations and patterns of normalization in both resuscitation methods used.

***Institutional Protocol Number*:** GZ0064.11/3b/2011

## Introduction

Despite intensive efforts to optimize outcome after cardiac arrest (CA) further improvements are urgently needed.[1–4] We hypothesize that neurological injury, caused by cerebral ischemia and reperfusion[5–7] can be mitigated by use of improved resuscitation methods. Extracorporeal life support (ECLS), a hybrid modification of cardiopulmonary bypass (CPB) and extracorporeal membrane oxygenation (ECMO), became an option to potentially increase the chance of survival and facilitate full neurologic recovery. Several studies have been published suggesting potential beneficial effects of ECLS compared to standard cardiopulmonary resuscitation (CPR).[8–11] Studies comparing these different measures of resuscitation during and after global ischemia by investigation of parameters obtained directly from the target area have not been published so far.

Cerebral ischemia and reperfusion injury caused by CA are complex processes and attempts have been made to elucidate the pathophysiological effects of extracorporeal cardiopulmonary life support (ECLS) on reperfusion injury, but many questions remain to be answered. Previous animal studies characterized neuronal death as an excellent target to evaluate efficacy of therapies in cardiac arrest. But neurological and functional outcome after cardiac arrest in rodents are challenging subjects and require long-term survival.

Since the CA1 region of the hippocampus showed a pronounced vulnerability to the ischemic conditions of CA it seems to be a suitable target area for measuring markers of cerebral hypoxia as a surrogate for hypoxic damage in cardiac arrest and reperfusion.[12]

Microdialysis (MD) is a minimally-invasive sampling technique, used for continuous measurement of free, unbound concentrations in the extracellular fluid of virtually any tissue.[13] Experimental CA arrest studies, using MD sampling, have reported dynamic changes in cell damage sensitive parameters such as glutamate. These changes were evident during circulatory standstill as well as in resuscitation and suggested a correlation between macro-circulation in the targeted area and these measurements.[14] Markers measuring cerebral ischemia caused by cardiac arrest and consecutive resuscitation, and reflecting the metabolic changes after successful resuscitation are urgently needed to enable a more personalized resuscitation and post resuscitation care.

Aim of this study was to investigate the metabolic changes in the hippocampal CA1 region during VF-CA and resuscitation with ECLS or conventional chest-compression CPR.

## Methods

This multistep study protocol was approved by the Institutional Animal Care and Use Committee of the University of Vienna and the Austrian Ministry of Science, Research and Economy (Protocol number: GZ0064.11/3b/2011). Experiments were conducted in compliance with EU regulations for animal experimentation (Directive 2010/63/EU of the European Parliament and of the Council) and reporting is in accordance with current ARRIVE guidelines. Healthy, wild-type, drug-naïve, male Sprague-Dawley rats (n = 30; Himberg Austria; 350±50g) were brought to the laboratory 14d before the experiment, maintained on 12:12h light/dark circle with ad libitum access to water and food and were adapted to the new environment. Animals were housed in cages with 1–3 companions. Welfare related assessment was performed on a regular basis by animal technicians and two veterinarian physicians. Animals were monitored continuously until brought to the facilities, then at 8, 12, 16 and 24 hours for signs of distress, pain and general and social behaviour. Every 24 hours additionally weight was measured and teeth status was obtained. A detailed protocol for post operative animal management, established by veterinarians, animal technicians and research fellows is provided (S1 Appendix). No animals were euthanized before the endpoint.

### Microdialysis probe placement

Three days prior to the cardiac arrest experiment implantation of the cerebral MD guiding cannula (CMA 11 guiding cannula, CMA, Stockholm, Sweden) was performed. Thirty adult, male Sprague-Dawley rats (350-550g; Himberg, Austria) were anesthetized with sevoflurane 6% (Sevorane, Aesica Queensborough LTD, UK) for 5min Piritramid (0.3mg/100g, Hameln Pharma, Germany) and Caprofen (5mg) was administered to provide adequate analgesia. The animals were intubated with an adapted peripheral venous cannula (14GA venflon™ BD Luer-LokLock™, Helsingborg, Sweden) and mechanically ventilated at a rate of 65/min, with a volume of 6ml/kg, at FiO2 of 0.5 (Havard^®^ Inspira advanced safety ventilator, volume controlled, MA1 55–7058, Holliston, MA, USA). Ventilation rate was adapted to maintain an end tidal CO2 concentration of 35-45mmHg. To maintain anesthesia during preparation sevoflurane admixture was set to 3.5%.

After median incision of the skin the landmarks of the calvaria (Bregma, Lambda) were assessed. According to ‘The rat brain atlas’ [15] coordinates for probe placement (-3.96mm posterior and -1.4mm lateral right of the Bregma) were marked using a stereotactic frame [Harvard Dual Lab Standard Stereotaxic Frame for Rats, Harvard Apparatus, Holliston, MA, US] and a hole, with a diameter of 0.5mm was drilled in the calvaria, using a hand drill [Micromot 50/E, Proxxon, Föhren, Germany]. The MD guiding cannula was stereotactically inserted to rest in a tip-depth of 2mm below surface, allowing the MD membrane later to protrude exactly into the CA1 region of the right hippocampus [‘The rat brain atlas’ [15] Figure 66]. After placement of 2 micro screws in the left frontotemporal calvaria the guiding cannula was fixed using dental cement [Harvard dental international GmbH, Hoppegarten, Germany]. After cessation of sevoflurane animals were weaned from the respirator and upon confirmation of sufficient spontaneous breathing animals were extubated and brought back to the facilities. Post-procedural analgesia was performed by a combination of Piritramid (0.3mg/100g/BW subcutaneously, up to 3 times per day) and Caprofen (5mg subcutaneouly, once daily) and repeated as necessary. Animals had free access to food and a piritramid/water mixture (250ml H_2_0, 10ml glucose 33% and 30mg piritramid). In two animals initially allocated to standard CPR treatment, a technical defect resulted in premature experiment termination. These two animals were excluded from the study.

### Experiment

After three days animals were randomly allocated to one of the three groups: ECLS (n = 8), CPR (n = 18) or Sham (n = 2). Animals were anesthetized with initial sevoflurane 6% (Sevorane, Aesica Queensborough LTD, UK) for 5min and continuous sevoflurane 3.5%. Piritramid (0.3mg/100g, Hameln Pharma, Germany) and Caprofen (5mg) were administered to provide adequate analgesia. Animals were intubated (14GA venflon™ BD Luer-LokLock™, Helsingborg, Sweden) and mechanically ventilated (Havard^®^ Inspira advanced safety ventilator, f 65/min, 6ml/kg, FiO2 0.5, Holliston, MA, USA) providing necessary adaptions to maintain an end tidal CO2 of 35-45mmHg.

Subcutaneous 3-lead Electrocardiogram (ECG) (Polygraph, Grass Instruments, Quincy, MA), rectal (Trec) as well as esophageal (Tes) temperature probe (General Purpose Sensor 9F, Mon-a-therm™, A Mallinckrodt Company, Mexico) were inserted. Baseline Tes was maintained at 37±0.2°C using an operating table for small animals (Medax GmbH & Co, Neumünster, Germany) and a neonatal heating lamp (Manual control infant warmer, Fisher&Paykel Healthcare, New Zealand).

**Fig 1:**
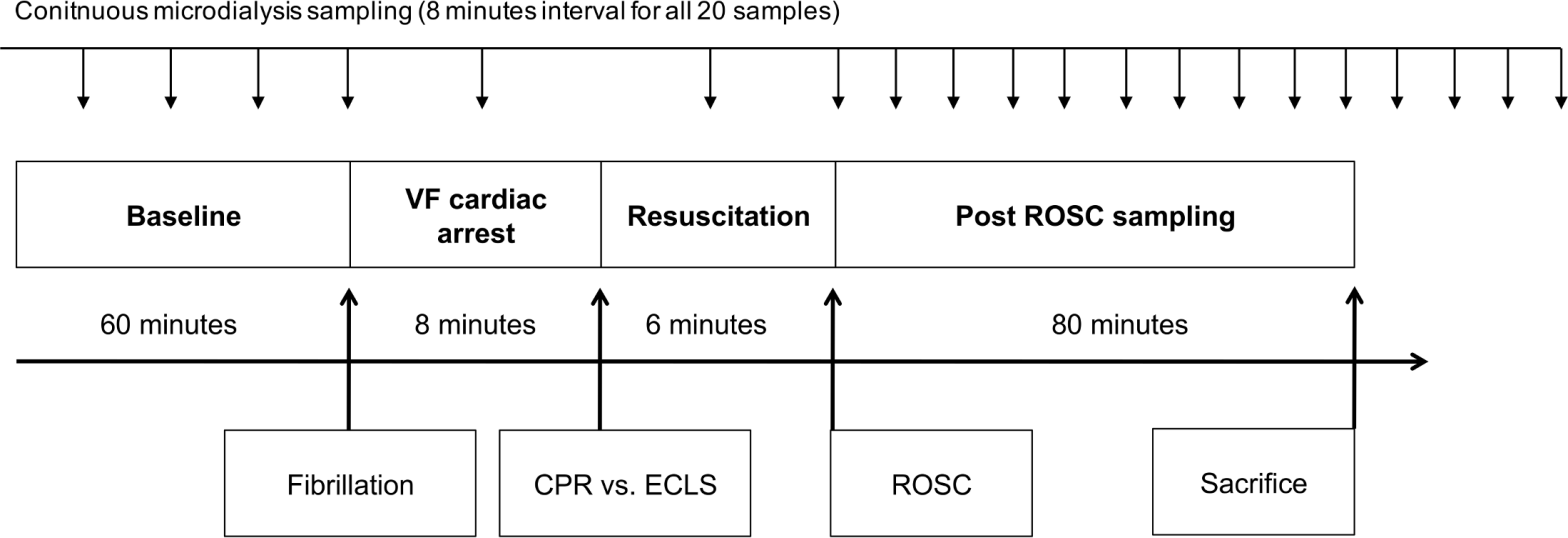
Timeline of the experiment. Microdiyalsis sampling interval conituously with 1 sample over 8min; Time scale is non linear to allow better overview; VF, ventricular fibrillation; ROSC, return of spontaneous circulation; CPR, cardiopulmonary resuscitation; ECLS, extracorporeal cardiopulmonary life support.

After preparation and distal ligation, a 2.5Fr neonatal umbilical venous catheter (Tyco healthcare, MA, USA) was inserted into the left femoral vein and artery (advanced in vein 9cm, in artery 6cm) for application of drugs and fluids, arterial blood gas analysis (BGA; measuring pH, paO_2_, paCO_2_, lactate and hemoglobin) and continuous mean blood pressure measurement (MAP). Before start of experiment 2 baseline samples of arterial BGA were taken. The timeline of the experiment is displayed in Fig 1.

### ECLS group (S1 Fig)

The ECLS experiment was based on the published rodent model.[12,16] After administration of 500 IU/kg of unfractionated Heparin, cardiopulmonary bypass cannulas were inserted into the right femoral artery (20- gauge Angiocath, Becton Dickinson, Sandy, UT) and the right jugular vein (five-hole 14-gauge venous cannula). A neonatal pacing catheter (Vygon GmbH, Aachen, Germany) was inserted through the jugular cannula to apply electric current directly to the right ventricular endocardium.

Sevoflurane was stopped 90 seconds before VF-induction. Ten seconds after cessation of mechanical ventilation, VF was induced by a 2min impulse of 12V/50 Hz alternating current and ensured by ECG readings and reduction in MAP. If spontaneous defibrillation occurred, additional impulses were delivered for 30 seconds. Afterwards the animal remained untouched to achieve a total of 8 min of untreated CA.

Epinephrine 20 μg/kg, and heparin 100 IU were added to the ECLS-reservoir. At 8-min VF-CA, ventilation (f 20/min and FiO2 1.0) and ECLS (Bloodflow 30 mL/min, gasflow 100ml/min, 100% Oxygen) was started. Blood flow was increased up to 100 mL/kg/min At 75 seconds of ECLS, epinephrine 10 μg/kg was administered intravenously. At 2 min of ECLS, defibrillation attempts were started (5J, monophasic), with defibrillation series of up to three shocks if ventricular fibrillation or non-perfusing ventricular tachycardia was present. Animals were weaned from ECLS and the jugular venous bypass cannula was removed 2min after ROSC, if MAP was 50 mmHg or above.

### Experiment—CPR group (S2 Fig)

The neonatal pacing catheter was implanted via the right jugular vein, which was ligated cranially to the insertion before placement. VF-CA-Induction was performed as described above.

Epinephrine 20 μg/kg, and heparin 100 IU were used 10 seconds before the start of CPR and 75 seconds after start of CPR another bolus of epinephrine 10 μg/kg was administered. At 8 min of VF-CA, ventilation (100% oxygen at a ventilation rate of 20/min) and chest compressions with a hydraulic chest-compression-device (Streubel Automation, Grampersdorf, Germany; at a rate of 200/min) were started. After 2 min of CPR, defibrillation was attempted if feasible (5J, biphasic), with a defibrillation series of up to three shocks if unsuccessful. Similar to the ECLS group this algorithm was followed for a maximum of 10min from the start of resuscitation attempts.

### Post ROSC period–all animals

The occurrence of ROSC was defined as spontaneous heart beat with pulsatile arterial blood flow and MAP ≥50mmHg for 5min. Ventilator parameters were changed to pre CA settings as soon as ROSC was achieved. Fluid boluses of 0.1ml were applied intravenously to maintain a MAP of at least 50mmHg. Maximum fluid balance was +10ml. Inotropic agents and vasopressors were not administered. Animals without hemodynamic stability under those measures were sacrificed. Temperature management after ROSC was performed using the same measures as before to maintain 37±0.2°C during the whole post resuscitation period. If there was no ROSC after 10min the experiment was ended and sampling was performed for 32 min to obtain post mortem samples.

### Sham procedure

Intubation, analgo-sedation and monitoring as well as surgical procedure were identical to the CPR group. Without inducing cardiac arrest, the same sampling intervals were chosen.

### Necropsy

Upon completion of sampling, animals were sacrificed, brains were removed and fixed in 7.5% buffered formaldehyde solution. Coronary sections of 2 mm thickness containing the site where the MD probe had been inserted into the brain were embedded in paraffin wax. Serial sections of the entire paraffin blocks were cut at 2μm thickness. Of the resulting sections every 50^th^ section was mounted on glass slides and stained with Haematoxylin and Eosin (H&E). These sections were evaluated by means of light microscopy to determine the position of the MD probe and side effects due to probe implantation. To assess neuronal damage due to cardiac arrest in animals that achieved ROSC the following selectively vulnerable brain regions were evaluated in H&E-stained sections: cerebral cortex, the CA1 region of the hippocampus and the thalamic reticular nucleus.

### Microdialysis probe sampling and evaluation

The MD circuit consisted of a two-syringe pump (SP101i, WPI, Shanghai, China), two 37.5 cm in- and outflow tubing systems and a CMA 11 MD probe. The circuit was perfused with artificial cerebrospinal fluid (Perfusion Fluid CNS, CMA, Stockholm, Sweden) at a flow rate of 1μl/min After endotracheal intubation of the animal, the MD probe was placed into the guiding cannula. A run-in phase was performed until the end of the surgical procedure (but at least for one hour), to allow equilibration after the insertion trauma. A sampling interval of 8min was chosen to achieve a high temporal resolution. Prior to the induction of VF four baseline measurements were performed. After VF induction a maximum of 12 samples were collected. Four additional samples were collected after the animals had been sacrificed using a bolus injection of 5mmol of potassium (or after death due to hemodynamic instability). Microvials were centrifuged and analysed for lactate, pyruvate, glutamate and glucose using a CMA 600 analyser (CMA, Stockholm, Sweden / M dialysis AB, Johanneshov, Sweden).

### Statistics

Continuous data are presented as median and 25% to 75% interquartile ranges or mean with standard deviation if appropriate. Categorical data are presented as count and relative frequency. MD results are presented as obtained concentrations without correction for recovery. Recovery experiments and development of the setup according to optimal in vitro conditions are reported elsewhere.[17] Sample size calculation was performed with main target value L/P ratio, alpha error of 0.05, power of 80% an estimated effect of 70% and 50% standard deviation, resulting in an initial sample size of 16 animals. This simple size was increased after the original series to achieve an equal sample size post ROSC in the two intervention arms, resulting in a total sample size of 8 animals in the ECLS and 18 animals in the CPR group.

To obtain lactate/pyruvate ratios the appropriate values were calculated for each animal at each time point. Differences between the outcome groups were assessed using students t-test and non parametric testing for continuous variable and chi-square test for binary variables. For data management and analyses we used MS Excel 2008 for Mac (Microsoft, Redmond, CA), SPSS Statistics 20 (IBM Corp., Armonk, NY). The statistician performing the analysis was blinded to experimental group status. Generally, a two-sided p-value <0.05 was considered statistically significant.

## Results

Ventricular fibrillation CA was successfully induced and maintained for 8 min in 26 intervention-animals. After a median resuscitation time of 6 min (IQR: 4;8) and 3 defibrillation series 10 animals (39%) achieved ROSC. More than twice as many animals achieved ROSC in the ECLS group compared to the CPR group with 5/8 vs. 5/18 animals (OR 4,3; 0.7–25; p = 0.189).

Baseline hemodynamic parameters and results of the baseline arterial BGA are displayed in Table 1.

**Table 1:** Baseline characteristics.

	ECLS^a^ (N = 8)	CPR^b^ (N = 18)	SHAM (N = 2)
Esophageal temperature C°	37.0 (±0.1)	37.0 (±0.1)	37.0
ABP^c^—mmHg	76 (±17)	77 (±13)	75
Baseline pH	7.404 (±0.04)	7.395 (±0.05)	7.370
Baseline pO_2_ mmHg	203 (±33)	172 (±67)	158
Baseline pCO_2_ mmHg	41 (±4)	42 (±5)	42
Baseline lactate mmol/l	1.9 (±0.7)	1.6 (±0.4)	1.3
Baseline Hb^d^- g/dl	12.7 (±1.0)	13.6 (±1.2)	12.4

Values are displayed as mean ±standard deviation

^a^ECLS, extracorporeal cardiopulmonary life support

^b^CPR, cardiopulmonary resuscitation

^c^ABP, arterial blood pressure

^d^Hb, hemoglobin level

There was no statistical significant difference between the study groups’ baseline parameters.

**Fig 2:**
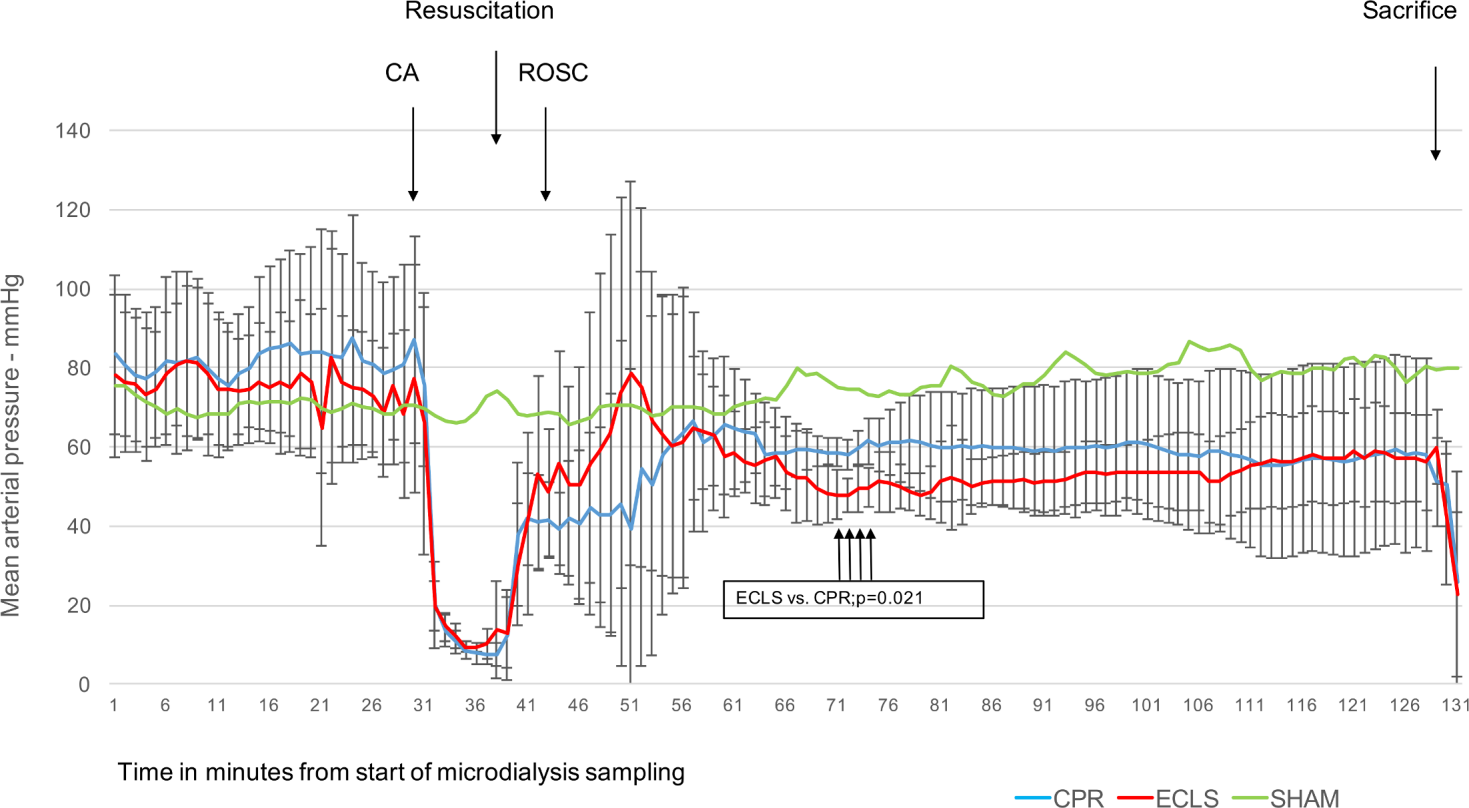
Mean arterial blood pressure in mm Mercury against time. X-axis: time in minutes; Y-axis: mean arterial blood pressure invasively measured in mmHg (mean with standard deviation); VFCA, ventricular fibrillation cardiac arrest; ROSC, return of spontaneous circulation; ECLS, extracorporeal life support; CPR, cardiopulmonary resuscitation; The p value displayed results from comparison of mean values the interval of measurements min 70 to 74.

Mean arterial blood pressure (MAP) values during the course of the experiment in the two intervention groups and the Sham animals are shown in Fig 2.

Animals achieving ROSC after CPR showed higher MAP values after 21min after ROSC for a period of 4min. After successful resuscitation the BGA showed significantly lower hemoglobin (8g/dl vs. 12g/dl; p = 0.042), lower glucose (13mmol/l vs. 20mmol/l; p = 0.012) lower lactate (9mmol/l vs. 14mmol/l; p = 0.042) and lower potassium levels (4.5mmol/l vs. 6mmol/l; p = 0.025) in the ECLS group, most probably reflecting hemodilution caused by the priming fluid.

Central nervous system MD probes were successfully installed in all 28 animals. Correct position of the MD probe in the target area was verified, as described above in 23 of 27 animals (S3 Fig). In one animal probe position could not be evaluated due to technical problems and four were slightly off target. Implantation of the guiding cannula led to mild mononuclear infiltration of the meninges, superficial edema, and gliosis in the cerebral cortex (S4 Fig). Bone fragments were frequently displaced into the neuropil. Implantation of the MD probe led to mild bleeding in most animals, however in four animals severe bleeding in the white substance adjacent to the hippocampus and in the lateral ventricle was evident.

**Fig 3:**
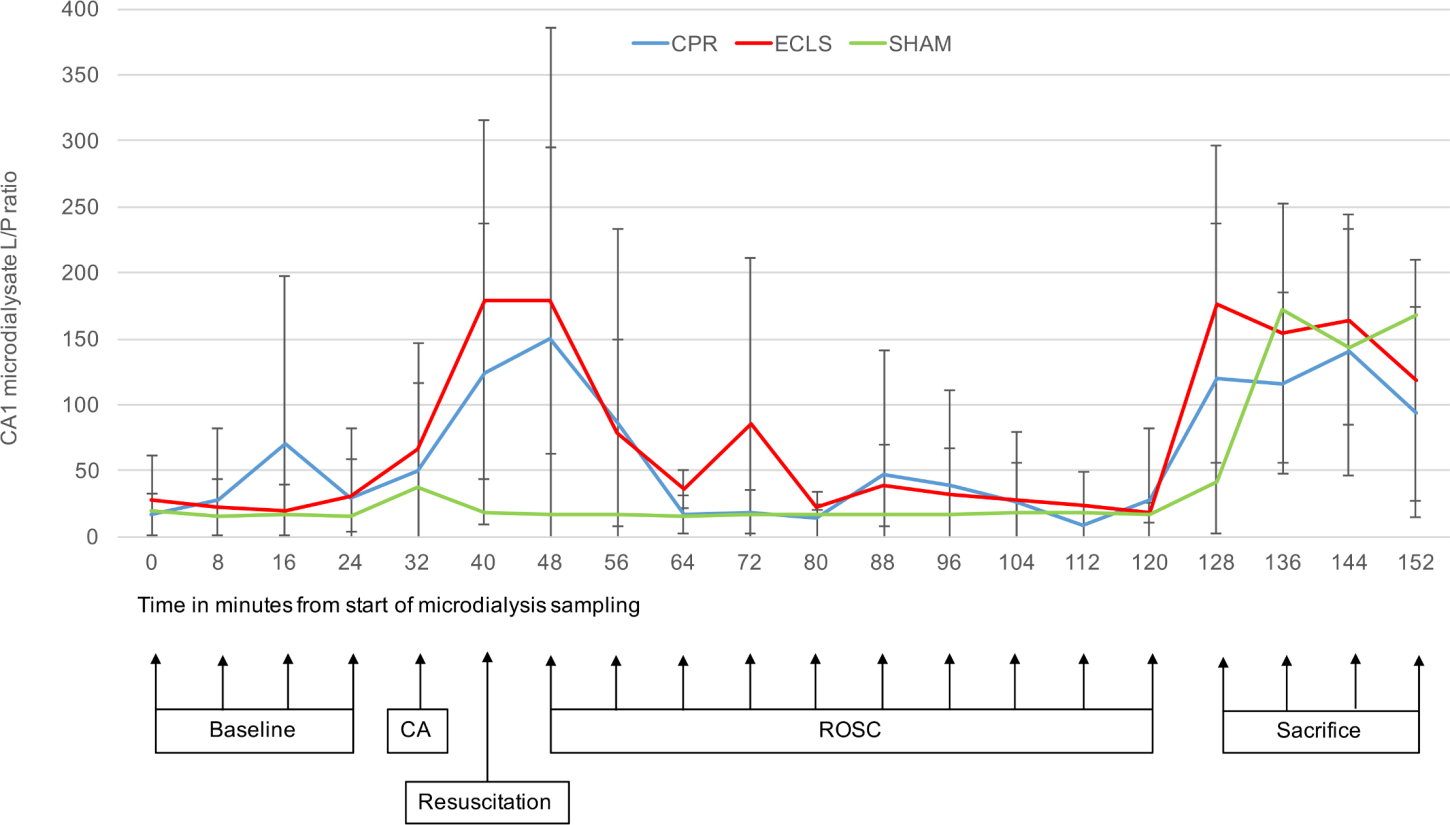
CA1 lactate/pyruvate ratio against time. x-axis: Point of measurement, measurements 8min apart (sampling interval of 8min); y axis: Lactate/pyruvate ratios (mean values and standard deviation); CA, cardiac arrest; CPR, cardiopulmonary resuscitation; ECLS, extracorporeal cardiopulmonary life support; BL, baseline; ROSC, return of spontaneous circulation.

**Fig 4:**
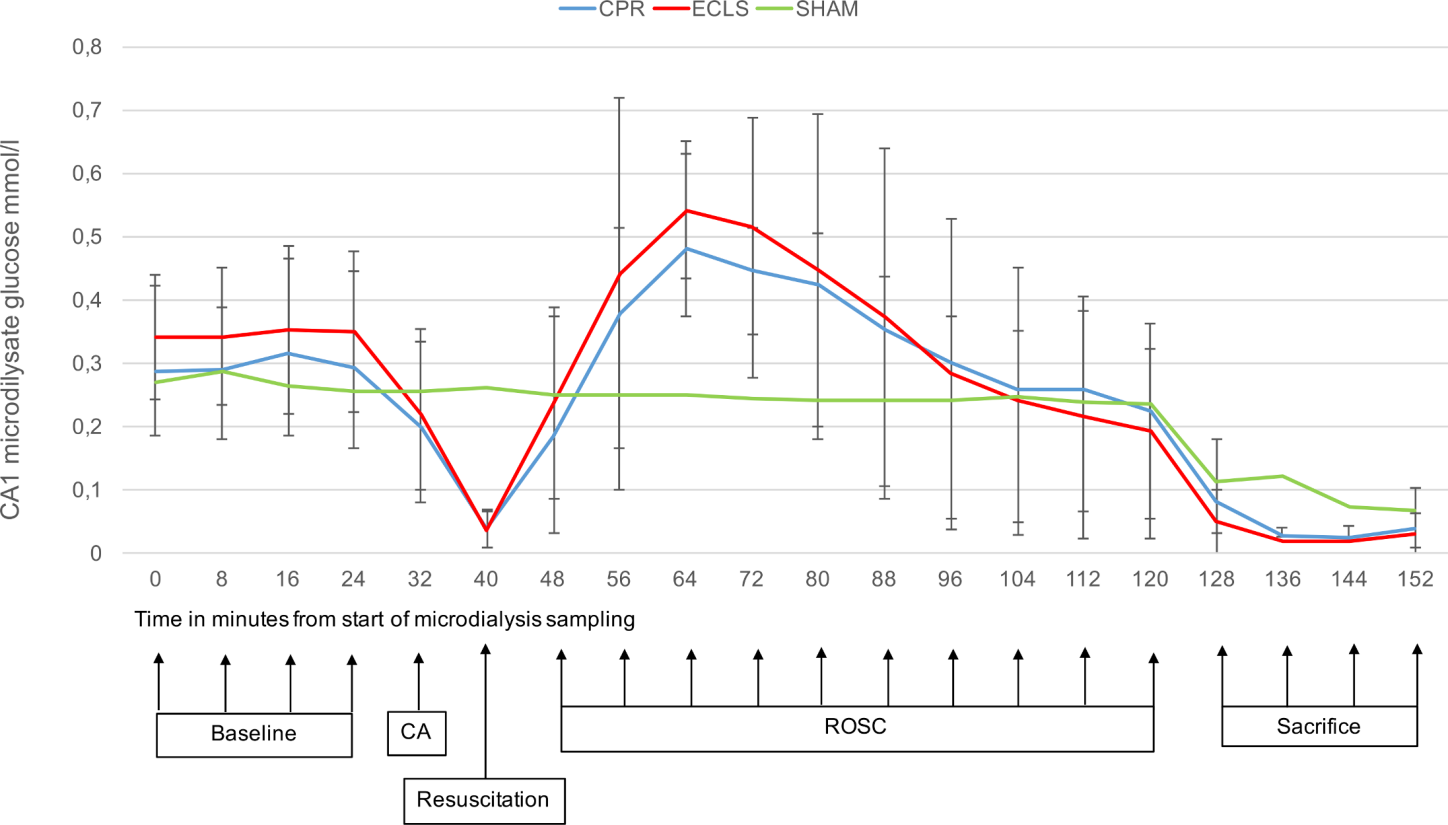
CA1 glucose mmol/l against time. x-axis: Point of measurement, measurements 8min apart (sampling interval of 8min); y axis: Glucose (mmol/l; mean values and standard deviation); CA, cardiac arrest; CPR, cardiopulmonary resuscitation; ECLS, extracorporeal cardiopulmonary life support; BL, baseline; ROSC, return of spontaneous circulation.

**Fig 5:**
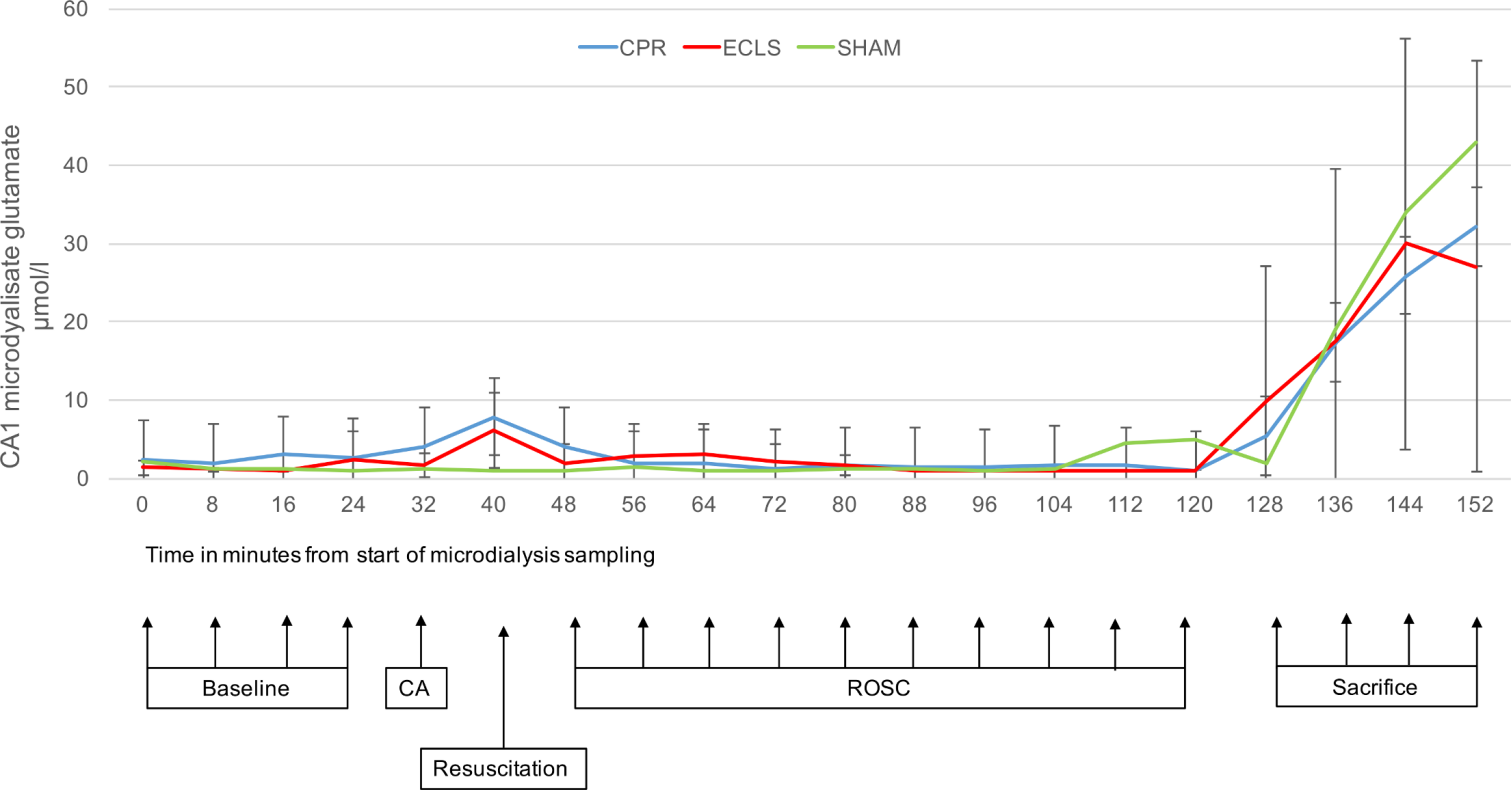
CA1 glutamate μmol/l against time. x-axis: Point of measurement, measurements 8min apart (sampling interval of 8min); y axis: Glutamate (μmol/l; mean values and standard deviation); CA, cardiac arrest; CPR, cardiopulmonary resuscitation; ECLS, extracorporeal cardiopulmonary life support; BL, baseline; ROSC, return of spontaneous circulation.

All measured parameters showed a stable baseline before induction of CA. Values for lactate/pyruvate ratio, glucose and glutamate and their changes during the experiment are displayed in Figs 3–5.

After induction of CA there was a significant difference in all measured parameters compared to baseline and Sham animals. After ROSC all values returned to baseline levels.

Central nervous glucose concentration showed a significant decline after the induction of CA with its minimum during resuscitation (0.32±0.1mmol/l to 0.04±0.01mmol/l; p<0.001) and a significant rise (to 0.53±0.1 mmol/l; p<0.001) compared to baseline values after successful measures of resuscitation of any kind. After peaking, 24min post ROSC, a return to the baseline values was observed, in both resuscitation groups at 64min after ROSC. There was no difference between the methods of resuscitation.

Lactate/pyruvate (L/P) ratio showed a 5fold increase (31 to 164; p<0.001) after induction of CA and reached its maximum at 8min post ROSC. In animals, which achieved ROSC, a decline in L/P ratio towards the baseline values was observed after successful resuscitation. L/P ratio in intervention animals did not differ significantly from Sham values 24min after ROSC. There was no difference between the methods of resuscitation.

Glutamate values showed a 3.5-fold increase to (2.06 to 7.12±5.1μmol/L; p<0.001) in early resuscitation before ROSC. In animals, which achieved ROSC the concentrations quickly resembled baseline values. There was no difference between the methods of resuscitation. The increase, which occurred in all animals after sacrifice until end of measurements was substantially larger compared to values measured in CA and resuscitation.

**Fig 6:**
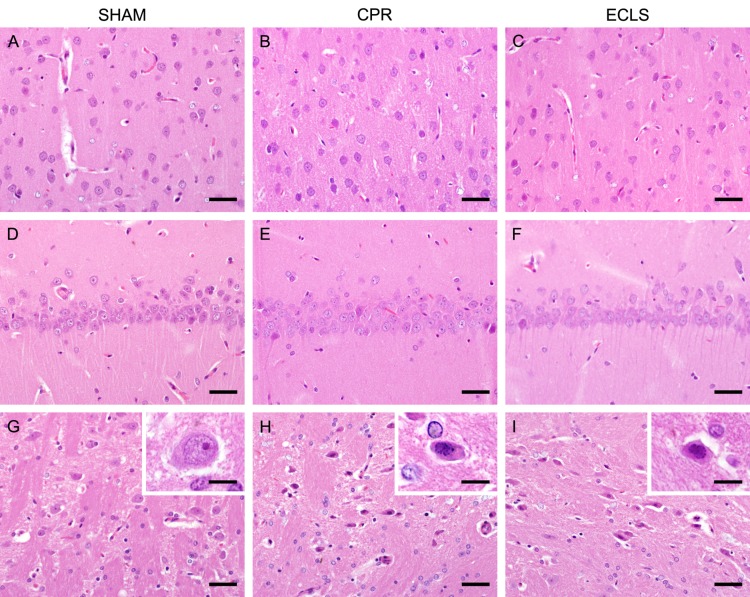
Representative pictures of cerebral cortex, hippocampal CA1 region and thalamic reticular nucleus. A-C: Cerebral cortex without lesions in sham (A), CPR (B) and ECLS (C) animals; H&E staining, bar = 40μm; D-F: Hippocampal CA1 region without lesions in sham (D), CPR (E) and ECLS (F) animals; H&E staining, bar = 40μm; G-I: Thalamic reticular nucleus, no lesions in sham animal (G), numerous shrunken neurons with condensation of nuclear chromatin in CPR (H) and ECLS (I) animals; H&E staining, bar = 40μm; inserts show close-up views of one neuron in the respective animal; H&E staining, bar = 10μm.

In animals that achieved ROSC selectively vulnerable brain regions were evaluated in H&E-stained sections. Neither sham animals nor animals subjected to cardiac arrest regardless of the resuscitation methods showed lesions in the cerebral cortex or the CA1 region of the hippocampus (Fig 6A–6F). In the reticular thalamic nucleus of sham animals no damage was evident (Fig 6G), but animals of the CPR-group and the ECLS-group showed early nuclear damage. Neurons were slightly shrunken with condensation of nuclear chromatin (Fig 6H–6I). These alterations were detectable in numerous neurons of the reticular thalamic nucleus in all animals of the CPR-group and all but one animal of the ECLS-group, which showed only few degenerated neurons.

## Discussion

In a rodent model, ischemia and resuscitation associated changes can be displayed almost in real time using MD in the hippocampal CA1 region during VF-CA. Severe alterations in ischemia-sensitive markers (glucose and lactate/pyruvate ratio) after the induction of cardiac arrest, reflecting global cerebral ischemia, could be observed. During resuscitation these parameters showed significant changes with similar patterns in both used resuscitation strategies (CPR and ECLS). The rate of ROSC was descriptively higher in the group resuscitated by cardiopulmonary bypass, but the results did not reach significance, presumably because of the small sample size. The rate of ROSC in both groups and the ratio of ROSC between ECLS and CPR are comparable to previously published results.[12]

Microdialysis parameters showed a global hypoxic pattern, reflected by an increase in L/P ratio and glutamate after induction of CA and a significant decline of the remaining glucose during CA probably because glucose is metabolized in the CNS while further supply is decreased due to cessation of blood flow. Interestingly glucose values spiked significantly after resuscitation, probably due to compensatory mechanisms caused by elevated peripheral blood glucose levels and restored circulation and the iatrogenic effect of epinephrine. Alternatively it could be caused by an inability of the damaged neurons to metabolise glucose. Likewise elevated blood glucose levels can be observed in clinical post cardiac arrest situations and have been linked to duration of CA and outcome.[18,19] Increase in L/P ratio after induction of ischemia reflects anaerobic glycolysis and normalisation after resuscitation seems to correlate with restoration of oxygen dependent energy metabolism. The stable course of glutamate, which showed only a slight increase after induction of CA and a fast normalization afterwards seems to reflect that hypoxic stress was induced, but cytotoxicity leading to glutamate release did not occur during the experiment.[20] An alternative explanation could be, that the glutamate release triggered by neuronal damage could have been re-shifted by astrocytes in the glutamate-glutamine cycle.

The changes of all MD parameters represented the anticipated hypoxic response and specifically the glutamate rise after the circulatory standstill was in accordance to previously published data of cardiac arrest with conventional resuscitation.[14,21,22] Since data of metabolic changes during global central nervous system ischemia with the use of ECLS have not been published so far it is not possible to compare these findings to other studies.

Regarding our experience with ECLS and its superiority in achieving ROSC and the reported favourable outcomes in experimental and clinical settings we would have expected it to be different compared to CPR in terms of cerebral perfusion. Comparing our model to the reality, in a clinical setting, an important difference has to be noted that might explain, why our measurements could not demonstrate the suspected difference. In our rat model, very short durations of resuscitation (up to 10 min) were used, while in the clinical setting ECLS is primarily reserved for prolonged resuscitation efforts, after conventional CPR failed. It is known that coronary and cerebral perfusion pressures decline during prolonged experimental resuscitation with conventional CPR, whereas ECLS can sustain viability of the organism for longer time periods.[23]

Surprisingly the animals achieving ROSC after conventional cardiopulmonary resuscitation showed higher mean arterial blood pressure for several minutes after ROSC (Fig 2) compared to the ECLS group. This difference, however did not seem to influence the markers evaluated by MD in the target region of the hippocampal CA1 region. This leads to the question if measures of macro perfusion, such as arterial blood pressure are sufficient surrogates for oxygen delivery to the most sensitive target area, the brain. In clinical practice these parameters are used to feedback resuscitation efforts, but more refined technics might be valuable to guide our treatment strategies in the future.

None of the animals that achieved ROSC and survived for 80 min showed lesions in cerebral cortex or hippocampal CA1 region histologically. This is consistent with delayed cell death known in these selectively vulnerable brain regions in animal models and humans.[14,24–27] In contrast neurons in the thalamic reticular nucleus showed early degenerative change in rats that underwent CA irrespective of the resuscitation method used. This was unexpected, although early neuronal injury has been reported in this nucleus. However, survival times in these rat models had been at least six and four hours, respectively.[25,28] We therefore conclude that eight minutes of CA were sufficient to induce consistent neuronal damage in our model.

Our study was a controlled study with a fairly small sample size. Due to the low rate of ROSC in the standard CPR group we increased our sample size to achieve equal groups in both intervention arms after ROSC. Furthermore, we are aware of hemodynamic differences in the two intervention groups as animals conventionally resuscitated showed significantly higher MAP after ROSC for a short period of time. In our experience these differences have not been predictive for outcome and were therefore not altered, aiming to keep additional interventions to a minimum. In the literature the cerebroprotective effect of anesthesia, specifically sevoflurane pre- and postconditioning is well described.[29] In our study this effect was not evaluated. However, it is unlikely for this effect to make a difference between the groups because all animals received the same agents.

The time to ROSC during CPR and ECLS could not be planned for and was caused by defibrillation success and consecutive stability. In some cases, a short time of post ROSC was therefore sampled during the cardiac arrest sampling period, resulting in a potential impact on the results of this one sample.

It has to be taken into consideration, that this very complex model enables a highly sophisticated evaluation of cerebral ischemia caused by VF-CA and during CPR and ECLS, which has never been preformed and published before.

## Conclusions

Metabolic changes during ischemia and resuscitation can be displayed by cerebral microdialysis in our VF-CA CPR and ECLS rat model. Microdialysis reflects a promising method to obtain parameters of perfusion and potentially steer future reperfusion and post resuscitation care. We found similar microdialysate concentrations and patterns of normalization in the hippocampal CA1 region in both resuscitation methods used.

## Supporting Information

S1 Appendix Detailed protocol for post operative animal management. (PDF)

S1 Fig Setup sketch of the ECLS experiment. (PDF)

S2 Fig Setup sketch of the CPR experiment. (PDF)

S3 Fig Correct position of the MD probe. Puncture channel in the hippocampal CA 1 region with mild bleeding; arrows: pyramidal layer of the hippocampus; H&E staining, 200x magnification.
(PDF)

S4 Fig Reactive brain lesions after implantation of the guiding cannula. Mild mononuclear infiltration of the meninges, edema and gliosis in the cerebral cortex; H&E staining, 100x magnification.
(PDF)
