# Supplementary material for: Microdialysis Assessment of Cerebral Perfusion during Cardiac Arrest, Extracorporeal Life Support and Cardiopulmonary Resuscitation in Rats – A Pilot Trial
Source: PLoS One. 2016 May 13;11(5):e0155303. doi: 10.1371/journal.pone.0155303 (PMC4866776; doi:10.1371/journal.pone.0155303)

Schmerz ist eine unangenehme sensorische und emotionale Erfahrung verbunden mit tatsächlicher oder potentieller Schädigung eines Gewebes oder die derart von betroffenen Personen beschrieben wird. Schmerz ist immer subjektiv! Die unangenehme sensorische oder emotionale Erfahrung kann vom Untersucher Mensch niemals vollständig nachvollzogen werden. Er kann nur nach eigenen Erfahrungen den Schmerz des Tieres begreifen. Eine angemessene Schmerzerkennung ist beim Tier oft schwierig! „Schmerzen“ und „erhebliche Schmerzen“ Tabellen. Bei vermuteten oder tatsächlich erkennbaren Schmerzen sowie während und nach schmerzhaften Eingriffen müssen Analgetika eingesetzt werden, es sei denn, dass dies mit dem Zweck des Tierversuchs nicht vereinbar ist. **Schmerzen, Leiden oder Schäden dürfen den Tieren nur in dem Maße zugefügt werden, als es für den verfolgten Zweck unerlässlich ist; insbesondere dürfen sie nicht aus Gründen der Arbeits-, Zeit- oder Kostenersparnis zugefügt werden.**

Leiden ist eine Befindlichkeit, die auf der Nichterfüllung der Grundbedürfnisse eines Individuums beruht. Der Begriff des Leidens verlangt einerseits keine andauernde oder gar nachhaltige Beeinträchtigung des Wohlbefindens. Andererseits beinhaltet er eine gewisse Erheblichkeit; danach bedeutet Leiden mehr als schlichtes Unbehagen, schlicht Unlustgefühle oder einen bloßen vorübergehenden Zustand der Belastung. Auch Leiden können nur durch eigene Erfahrungen oder Anschauungen beurteilt werden.

Schaden erscheint gegeben, wenn der Zustand, in dem sich das Tier befindet, zum Schlechteren verändert wird.

Bewusstsein: Eine Beurteilung der Reaktionsweise des Tieres auf verschiedene Umweltreize und damit des Zusammenspiels zwischen Sinnesorganen und Motorik. Änderungen im Normalverhalten (Ruhelosigkeit, Lethargie, Erstarrung, Krämpfe) werden entsprechend bewertet. Es wird das Verhalten der Ratten im Heimkäfig und beim Umgang mit dem Untersucher beobachtet. Ratten mit ungestörtem Bewusstsein erkunden neugierig ihre Umgebung, nehmen den Untersucher wahr und richten sich an der Käfigwand auf. Tiere, deren Bewusstsein reduziert ist, liegen meist isoliert von den anderen und zeigen eine verminderte Reaktion auf Umweltreize.
normal – ruhelos oder gedämpft – lethargisch – erstarrt - krampfend

Pflegeverhalten: Das Putzverhalten gibt Hinweis auf das Allgemeinbefinden des Tieres und stellt ein wichtiges Kriterium der emotionalen Reaktivität dar. Ratten verbringen einen Großteil der Zeit mit Körperpflege. Wenn das Putzverhalten eingeschränkt ist, erscheint das Fell struppig und glanzlos, die Augen und/oder die Nasenöffnungen sind verklebt. Das rötliche Sekret der Haderschen Drüsen, das von gesunden Tieren beim Putzen im Fell verteilt wird, sammelt sich zunächst im medialen Augenwinkel und verklebt später den gesamten

Augenbereich. Da es auch über den Tränennasenkanal abfließt, kann es auch zu rötlichem Nasenausfluss kommen. Ein vermindertes Pflegeverhalten gibt verlässlich Hinweis auf ein gestörtes Allgemeinbefinden, auf Schmerzen oder kann ein Anzeichen für eine eingeschränkte Motorik sein. Die Bewertung des Pflegeverhaltens erfolgt durch Inspektion.
vorhanden – nicht vorhanden

Laufen: Bewegungsabläufe gestört oder Bewegungsfähigkeit vermindert (Adduktion der Vordergliedmaße, Hypomotilität, Laufen im Kreis, Stehunfähigkeit). Ist die Bewertung im Heimkäfig nicht ausreichend, wird das Tier in einen offenen Käfig gesetzt, um den Bewegungsablauf beurteilen zu können. Ischämische Infarkte können gut erfasst und bewertet werden. Um zu prüfen, ob eine Adduktion der Vorderpfoten vorliegt, wird das Tier vorsichtig in Höhe der Schwanzwurzel ca. 1 cm über den Boden gehalten. Gesunde Ratten strecken die Vorderpfoten dem Boden entgegen. Tiere mit ischämischem Infarkt drehen beim Hochheben an der Schwanzwurzel ständig eine Vorderpfote auf die kontralaterale Seite der infarzierten Hemisphäre (von der einfachen Adduktion der Vorderpfote bis zum schraubenförmigen Ineinander Verdrehen des Tieres). Ischämische Infarkte können sich außerdem in einer Hypomobiltät äußern. Ein unilateraler Infarkt ist gekennzeichnet durch ein Laufen der Tiere im Kreis. Die Kreisbewegung erfolgt stets in Richtung der geschädigten Hemisphere. Dieser Test gibt Aufschluss über Motorik, Gleichgewicht und Stellreflexe der Ratten.
normal – Adduktion der Vorderpfoten – Hypomobilität – Laufen im Kreis - stehunfähig

Koma/OPC 4 die schwerste Form einer quantitativen Bewusstseinsstörung. In diesem Zustand kann das Individuum auch durch starke äußere Stimuli, wie wiederholte Schmerzreize, nicht geweckt werden. Das Koma ist somit ein Symptom (Krankheitszeichen) und keine Krankheit. Das Koma ist Ausdruck einer schweren Störung der Großhirnfunktion. Reflexe erhalten aber keine Reaktion auf verbale und/oder physische Kontaktaufnahme.

Bewusstseinstrübung/OPC 3 („Somnolenz/Sopor/Schlaf“) Erhaltung einer minimalen Form der Wahrnehmung, sodass eine Kontaktaufnahme möglich ist. Weck bar auf starke äußere Stimuli! Abhängigkeit von PflegerInnen für die notwendigsten Dinge des Lebens. Schwere Einschränkung der zentralnervösen Funktionen (Gedächtnislücken, Demenz, Lähmungen). Reaktion auf verbale und/oder physische Kontaktaufnahme!

Post operative Erholungsphase mit täglich erkennbarer Besserung des Bewusstseins und Genesung vom operativ gesetzten Schaden (z.B.: Lähmungen).

Kommunikation („mitteilen“) ist der Austausch oder die Übertragung von Informationen. Kommunikation ist alltäglich und verläuft scheinbar selbstverständlich, sodass sie nicht weiter problematisch erscheint. Für die meisten Situationen reicht dies auch aus! In unserem Fall

kann das allerdings nicht zwischen „Tür und Angel“ erfolgen sondern muss in „geoordneten Bahnen“ unmittelbar bei Auftreten des Problems laufen!

Richtlinien für Intensivbetreuung und Nachbetreuung im Stall:

1. **allgemeine Regeln**
    - **extrem** Zeitaufwendig, dazu ist sehr viel Geduld nötig
2. **Kontaktaufnahme**
    - Ansprechen
    - Körperkontakt
3. **Stall sauber halten**
4. **Handling**
    - am besten von unten, seitlich hingreifen
    - im Notfall am Schwanz packen
        i. Vorsicht bei Tieren über 600g da die Schwanzwurzel ausreißen kann
        ii. auf die Krallen muss man nicht so aufpassen
5. **Pflege**
    - *Augen*
        - wenn offen
            - Augensalbe
            - Verklebungen entfernen
            - mit feuchtem Tuch auswischen
            - bis sich das Tier selbst putzt
    - *Nase*
        - Ausfluss entfernen
    - *Zähne*
        - Kontrolle 1x in 24 h
        - bei Bedarf schleifen
        - nach 3–5 Tagen ohne feste Nahrung zu lang, müssen gestutzt werden, sonst kann das Tier nicht mehr fressen oder sie wachsen sogar ins Oberkiefer ein
        - Stutzen im Koma post ROSC oder in Narkose durch Wolfgang
    - *Zunge, Maul*
        - Befeuchten
    - *Lagerung*
        - Bequeme Druckfreie Position
        - Atemwege frei
        - solange die Tiere sich gar nicht bewegen können
        - darauf achten, dass die Nase frei liegt
        - Umlagern alle 8-12 h
    - *Massieren*
        - alle 8-12 h bis Ratte selbst bewegungsfähig
    - Blase
        - vorsichtig ausmassieren
    - Physiotherapie für Kreislauf

- **O_2**
  - so lange das Tier ruhig liegt
- *Umgebung*
  - alle 24 h Käfig mit neuer Betteinlage ausstatten

6. Medikation

- Vor und nach dem Spritzen Einstichstelle desinfizieren!
- *Injektion i.m.*
- *Injektion s.c.*
  zur Beruhigung und fürs bessere Handling Tuch über das Tier decken
- intravenös
- perkutan
- oral
- lokal

7. Systemische Analgetika [zur post-operativen Schmerzbehandlung, zB.: Wimmern (meist leise), struppiges Fell , verstärkter Rundrücken]

a) Opioide
  a.1. Buprenorphin, Piritramid, Pethidin, Butorphanol, Tramadol, Fentanyl
b) Nicht-Opioide
c) Nicht- steroidale Antiphlogistika (NSAIDs)
  c.1. Carprofen, Etodolac, Flunixin-Meglumin, Ketoprofen, Meclofenaminsäure, Meloxicam, Nifluminsäure, Phenylbutazon, Piroxicam, Tepoxalin, Tolfenaminsäure, Vedaprofen
d) Antipyretika
  d.1. Metamizol, Acetylsalizylsäure
e) Phenzyklidine
  e.1.1.Ketamin
f) Zur Anwendung kommen bei uns
  f.1. Piritramid/Dipidolor
  - stärkste Schmerzen aller Arten
  - Langzeitanwendung
  - Obstipation
  - Überwachung der Atemtätigkeit
  - Dosis (mg/kg):
    - Piritramid-Mix (15mg/5ml NaCl 0,9%):
      - 0,1ml/100g KG s.c. nach Bedarf und Nebenwirkungen
    - Piritramid-Trink'wasser' (30mg/250ml H2O + 30ml Gluc 5%)
      - 12-24h p.o.

  f.2. Carprofen/Rimadyl
  - Entzündungsschmerz
  - langfristig anwendbar
  - mit Opioiden kombinierbar
  - Dosis (mg/kg):
    - 4,0 oder 5,0 s.c./24h

8. Nahrungsaufnahme/Fütterung:

- Koma
  - subkutane Flüssigkeits-/Glukosezufuhr
  - alle 8-12 h

- Zwischenphase Koma/Somnolenz:
  - subkutane Flüssigkeits-/Glukosezufuhr & Füttern/Tränken
  - alle 8-12 h
- Somnolenz:
  - Füttern/Tränken
  - alle 8-12 h
- NaCl 0,9% sc:
  - anfangs prinzipiell verabreichen
  - wenn das Tier sich bewegt Wasserverbrauch dokumentieren und eher auf den Stress des Stechens verzichten und Feuchtfutter geben
  - Exsikkosezeichen Nackenfalte
- Wach
  - frisst selbst > alle 24 h
- Voraussetzung für Füttern und Tränken sind
  - spezielle Fütterungsmaßnahmen:
    - ✓ zB.: Verabreichung von aufgeweichten Futter in einer Schale auf dem Käfigboden
  - dass die Ratte selbständig schlucken kann und
  - dass die Notwendigkeit dafür gegeben ist wird festgestellt anhand
    - ✓ des Gewichtes
    - ✓ des Hautfaltenturgors
    - ✓ der Schleimhautfeuchtigkeit
    - ✓ beobachteter selbstständiger Nahrungsaufnahme
    - ✓ etc.
- Futter
  - Pellets sollten reichen
  - CaloPet-Paste oder Futtergelpads angefordert (folgt)

Pflegeprotokoll										
Versuchsdatum					Tiernummer					
**Tag**										
Datum										
**Stunde nach ROSC**	**08**	**12**	**16**	**24**	**Kommentare**	**08**	**12**	**16**	**24**	**Kommentare**
Uhrzeit (hh) [Uhrzeit für Visiten ±1h]										
Overall Performance Category (OPC 1-4)										
**Medikamente**										
NaCl 0,9% 1ml+ Gluc 5% 0,6ml (s.c. / 100g KG)*										
Piritramid-Mix 0,1 ml (s.c. / 100g KG)**										
Carprofen (Rimadyl) 0,4-0,5mg (s.c. / 100g KG)***										
**Pflege**										
Verklebungen von Augen und Nase entfernen****										
Augensalbe geben****										
Zunge und Maul befeuchten****										
Zahnkontrolle*****										
Umlagern****										
Physiotherapie/Massieren****										
Blase ausmassieren****										
neue Betteinlage										
Füttern*										
Wasser verabreichen*										
Unterschrift Untersucher										

*** bis Tier selbst frisst und trinkt / ** bis Tier selbst trinkt alle 8 h, öfter nach Bedarf / *** Tag 1 bis 3 alle 24 h, länger nach Bedarf / **** bis sich Tier selbstständig bewegt / ***** bis Tier hartes Futter frisst**

OPC 4: **Schwerste Form der quantitativen Bewusstseinsstörung. In diesem Zustand kann das Individuum auch durch starke äußere Stimuli, wie wiederholte Schmerzreize, nicht geweckt werden. Das Koma ist Ausdruck einer schweren Störung der Großhirnfunktion. Reflexe sind erhalten aber keine Reaktion auf verbale und/oder physische Kontaktaufnahme. Bewusstsein:** *erstarrt – krampfend* **Pflegeverhalten:** *nicht vorhanden* **Laufen:** *stehunfähig*

OPC 3: **(„Somnolenz/Sopor/Schlaf") Erhaltung einer minimalen Form der Wahrnehmung, sodass eine Kontaktaufnahme möglich ist. Weck bar auf starke äußere Stimuli! Abhängigkeit von Pflegerinnen für die notwendigsten Dinge des Lebens. Schwere Einschränkung der zentralnervösen Funktionen (Gedächtnislücken, Demenz, Lähmungen). Reaktion auf verbale und/oder physische Kontaktaufnahme! Bewusstsein:** *lethargisch* **Pflegeverhalten:** *nicht vorhanden* **Laufen:** *Laufen im Kreis - stehunfähig*

OPC 2: **Mäßige Schädigung, wach, ausreichende für das selbständige Überleben körperliche und zerebrale Funktion, wie zum Beispiel Nahrungssuche und Aufnahme. Kann aber noch Lähmungen, Krampfanfälle, Gangunsicherheiten, ungewöhnliche Lautäußerungen und Gedächtnisstörungen haben. Bewusstsein:** *ruhelos oder gedämpft* **Pflegeverhalten:** *vorhanden* **Laufen:** *Adduktion der Vorderpfoten – Hypomobilität*

OPC 1: **Gute annähernd normale körperliche und zerebrale Funktion. Bewusstsein:** *normal* **Pflegeverhalten:** *vorhanden* **Laufen:** *normal*

Visitenhäufigkeit: OPC 1: **24 stündlich /** OPC 2: **12 stündlich /** OPC 3-4: **8 stündlich**

Kriterien für die Verständigung eines verantwortlichen VersuchtiermitarbeiterIn um Entscheidungen über die weitere Vorgangsweise in Kooperation mit den MitarbeiterInnen am IBF zu treffen:

OECD OCDE

(For Display, or at Hand, in Animal Rooms and Facilities)

CLINICAL SIGNS AND CONDITIONS OF ANIMALS REQUIRING ACTION BY ANIMAL CARE STAFF AND STUDY DIRECTORS

Instructions:

When any of the following conditions or clinical signs are observed, the animal technician must immediately notify the responsible study director and/or veterinarian, and appropriate action should be taken. A decision should be made as to whether to humanely kill the animal, or to take other appropriate action to alleviate the pain and distress.

If there is a scientific necessity for not humanely killing or treating the animal(s) to alleviate the pain and/or distress, a written plan must be established indicating the schedule for future observations, and the decision endpoints or schedule for treatment or humane killing.

Clinical signs and conditions where humane killing may be appropriate:

1. **Any condition resulting in a prolonged or irreversible inability to eat or drink**, e.g., prolonged immobility, obstruction of the oral cavity, missing or abnormal teeth.
2. **Diseases or conditions indicating severe pain, distress or suffering**, e.g., fractures, self-induced trauma, abnormal vocalisation, abnormal posture or movements, open wounds or ulcers.
3. **Rapid or continuing weight loss**, e.g., 20% or greater body weight over a few days, or gradual but continued weight loss.
4. **Generalised decrease in grooming and abnormal appearance over an extended time period**, e.g., rough hair coat, extensive alopecia, prolonged diarrhoea, urine-stained hair coat, swollen limbs, paralysis, or other central or peripheral nervous disturbances (convulsions, circling behaviour, prostration).
5. **Severe or continuing respiratory distress**, e.g., coughing, sneezing, nasal discharge, bloody nares or mouth.
6. **Frank bleeding**, anaemia, or unusual discharges.
7. **Evidence of microbial infections or other diseases**, including those that interfere with the experimental protocol or cause any of the above.

For further details, see OECD Guidance Document: Recognition, Assessment and Use of Clinical Signs as Humane Endpoints for Experimental Animals Used in Safety Evaluation (OECD, 2000).

Bemerkungen/Beispiele zum Abbruch von Tierversuchen

Der Punkt, an dem Maßnahmen ergriffen werden sollen
basiert auf:

- Klinischen Anzeichen
- Pathophysiologischen Veränderungen
- Verhaltensveränderungen

Nicht alle im Folgenden vorgestellten Beispiele führen bei solitärem Auftreten sofort zum Abbruch des Experiments. Sie sind jedoch Anlass, das Tier dem Tierarzt vorzustellen, um gemeinsam über das weitere Vorgehen (Abbruch ja/nein, Änderung des Analgesieregimes, spezielle Pflegemaßnahmen, engmaschigere Überwachung u.a. Maßnahmen) zu entscheiden.

Beispiele für messbare Zeichen für den Abbruch
*(*wenn fehlende Besserungstendenz innerhalb von 3 Tagen*)*

- Körpergewicht -20%
- Körpertemperatur <30°C >40°C
- Herzfrequenz
- Atemfrequenz

Beispiele für parametrische Abbruchkriterien
*(*wenn keine Besserungstendenz innerhalb von 3 Tagen*)*

- struppiges, stark verschmutztes Fell
- geschlossene Augenlider
- mittel bis hochgradig abnorme Reaktionen auf einen Stimulus
- mittel bis hochgradig abnorme Körperhaltung
- klinische Krankheitszeichen wie Diarrhoe, Vomitus oder Dyspnoe
- Rektumprolaps
- Automutilation
- Bewegungsunfähigkeit

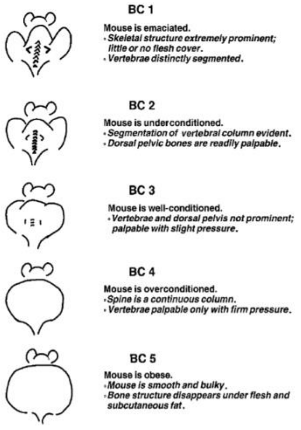

Body Condition Scoring (BCS)

1. sehr schlechte Kondition (ausgemergelt)
2. Skelett deutlich sichtbar
3. Verhältnis Skelett und Fleisch ausgewogen
4. Fleisch-Fettansatz überwiegt
5. zu viel fett

Gewichtsverlust

- wichtiger Hinweis auf anhaltende Schmerzen
- Rapider Gewichtsverlust von 20% innerhalb weniger Tage
  -> eindeutiges Abbruchkriterium
- gradueller Gewichtsverlust über eine gewisse Zeitspanne ohne Besserungstendenz
  -> Abbruch möglich bei Gewichtsverlust ab 20 %

Bewegungsunfähigkeit

- Tier erholt sich nicht innerhalb von 3 Tagen, sodass es nicht in der Lage aufzustehen und/oder sich fortzubewegen (Futter- & Wasseraufnahme erschwert bzw. nicht möglich)
- je nach Ursache & Schweregrad
  -> Abbruch
- Lähmungen nach operativen Eingriffen als Folge des Versuchsdesigns oft erst nach längerer Konvaleszenz reversibel!

Aufgaben Dienstmannschaft

- medizinische Betreuung der Versuchstiere
- sonstige Pflege der Versuchstiere
- Eintragen der Daten ins Versuchsprotokoll
- Erfassen der medizinischen Werte
- Alle mit einbeziehen, die mit den Tieren arbeiten, da jede Personengruppe einen unterschiedlichen Blickwinkel hat:
  - Tierpfleger kennen ihre Tiere
  - Tierärzte besitzen klinische Fachkenntnisse
  - Forscher kennen die potentiellen NW der Eingriffe & Behandlungen

Dienst

- ✓ Absprache mit diensthabendem Arzt/Tierarzt, Tierpfleger und/oder OP Personal
- ✓ Kontrolle: alle Medikamente vorhanden?
- ✓ Versuchsprotokoll und Datenblatt durchgehen
- ✓ Aufgaben erfassen
- ✓ ev. Zeitplan erstellen
- ✓ OP-Termin und Todesfälle auf Zetterl am Käfig dokumentieren
- ✓ am Wochenende ist nach 14 Uhr kein Tierpfleger mehr da
- ✓ Zettel auf tote Ratten um das vorzeitige Wegschmeißen zu verhindern
- ✓ tote Ratten werden nur 3 Tage im Kühlraum belassen - dann weggeschmissen!!
- ✓ Ratte erst bei Kühlraumtür ausstreichen, wenn sie entsorgt wird!

Ablauf Dienste

1) **alle 8-12h**
2) Zeit immer in Minuten (min.) oder Stunden (h) nach ROSC (Return of spontaneous circulation = Wiedereinsetzen des Kreislaufs)
3) Dokumentation laut Tabelle!
4) Umlagern
5) Maßnahmen ins Versuchsprotokoll eintragen
   - Bilanzierung/Körpergewicht
   - Verabreichte Medikamente
     - genau mit Dosierung u Zeitangabe ins Protokoll eintragen
6) Dokumentation
   - alles, was getan wird, wird dokumentiert

Score Sheet für Abbruch
<u>(bei mehr als 10 Punkten der 14 folgenden Punkte über eine Dauer von 3 Tagen)</u>

1. Allgemeinzustand/Neurologie
   - schlaffer Schwanz
   - mittel bis hochgradig beeinträchtigter Gang
   - mittel bis hochgradig beeinträchtigter Korrekturreflex
   - nicht besser werdende mittel bis hochgradig Paraparese der Hintergliedmassen
   - persistierende vollständige Lähmung beider Hintergliedmassen
   - moribund
2. Habitus
   - Abmagerung (siehe oben)
3. Atmung
   - frequent – verlangsamt
   - mühsam
   - pfeifend, schniefend, keuchend
4. Zustand der Körperdecke
   - mittel bis hochgradig schmutziges Fell
   - mittel bis hochgradiger Haarausfall
   - Sträuben der Haare
   - Dehydratation
5. Defäkation/Urinieren Kot:
   - hochgradig abnorme Mengen
   - hochgradig abnorme Häufigkeit
   - persistierende abnomrale Kotform, -feuchte, -geruch
   - persistierende Diarrhöe
6. Harn
   - nicht feststellbar
   - Polyurie
   - Hämaturie
7. Verhalten
   - mittel bis hochgradig teilnahmslos
   - mittel bis hochgradig hyperaktiv
   - unbeweglich
   - mittel bis hochgradig aggressiv
8. Reflexe
   - Pupillen verengt, erweitert
   - hochgradig gesteigerte Schreckreaktion
   - spontane Muskelzuckungen
   - keine Reaktion
9. Aktivität
   - persistierende abnormale Dauer und Häufigkeit
     1. der Phasen des Schlaf- und Wachrhythmus
     2. der Futter- und Wasseraufnahme
10. persistierende veränderte Fellpflegeaktivität
    - mittel bis hochgradige gesteigerte Pflege

- kratzen
- lecken
- Selbstbeschädigung/Beißen

11. persistierende abnorme Haltung
    - mittel bis hochgradige Rückenkrümmung
    - Kopf- und Nackenstreckung
    - mittel bis hochgradige „aufgezogene“ Bauchdecken,
    - mittel bis hochgradige Streckung des Körpers
    - Verlagerung des Körperschwerpunktes (z. B. nach vorne oder hinten)
12. persistierende abnorme Fortbewegung
    - mittel bis hochgradige Bewegungsunlust
    - mittel bis hochgradige Lahmheit
    - mittel bis hochgradige gestörte Bewegungsabstimmung
    - im Kreis laufen
    - mittel bis hochgradig anormale Stellung der Gliedmaßen oder Gewichtsverteilung
13. persistierende abnorme Lautäußerungen
    - Schmerz- oder Schrecklaute
14. Allgemeines
    - persistierendes hohes Fieber
    - persistierendes Zittern
    - persistierende Krämpfe
    - persistierende Zuckungen

- **Abbruchkriterien bzw. humane Endpunkte**
  - schonende Tötung des Tieres, wenn möglich unter Vollnarkose und mechanischer Beatmung mit Thorakotomie mit Spülung der Organe, wenn innerhalb von 72 h keine Anzeichen von Rekonvaleszenz erkennbar sind
- **Kritische Zeitpunkte für das Tier und den Versuch & dementsprechende Überwachungshäufigkeit**
  - Beurteilung des Befindens der Tiere
    1. mind. 1 x täglich
    2. 2 bis 3 x täglich
       je nach vorgeschrittener Bewußtseinseinschränkung bzw Lähmungsgrad
- **Spezielle Haltungs- und Pflegemaßnahmen**
- **Protokollierung des Gewichts u.a. Maßnahmen**

Basic neurological and physiological observational assessment:

Neurologic function will be assessed daily, using a neurologic deficit score (NDS; 0%=normal, 100%=dead) and an overall performance category score (OPC; 1=normal; 2=slight disability; 3=severe disability; 4=comatose; 5=dead) until a defined period after cardiac arrest for assessment of final OPC and NDS. The procedure follows the set up by Irwin,42 however modified to adapt for rats using the NBRP Rat Kyoto protocol (The national BioResource

Projekt fort the Rat in Japan; http://www.anim.med.kyoto-u.ac.jp/nbr/strainsx/FOB_menue.aspx). A battery of tests will be applied to reveal defects in gait or posture, changes in muscle tone, grip strength, visual acuity and temperature. To complete the assessment, vitally important reflexes are scored. In addition, during the manipulations, incidences of abnormal behavior, fear, irritability, aggression, excitability, salivation, lacrimation, urination and defecation are also recorded.

Post operative observations

(1) Home-cage measurements

(Observe the animals from outside the cages, taking care not to agitate them. Do not touch the animals.)

a. Body position:
   - S (sitting or standing): Sitting or normally standing
   - A (asleep): lying on side curled up or crouching
   - R (rearing): Standing on hindlimbs
   - H (hunchback): Back is rounded, even when walking, and animal appears to be holding stomach
   - L (lying): Lying on side, limbs in air
   - F (flattened): Animal is spread out with abdomen pressed to floor
   - C (catalepsy): Animal is in a cataleptic-like state; may be 1 of a variety of postures, but animal must maintain an unnatural posture
   - Note: Only an assessment of S, A or, if of a mild degree, R is considered normal.

b. Respiration:
   - Normal
   - Slightly incomplete/fast and shallow, bradypnea (breathing either fast and shallow or slow)
   - Moderately incomplete/rapid breathing, difficulty breathing (breathing very fast and shallow or very shallow and labored in appearance)
   - Severely incomplete/wheezing, breathing with mouth open (wheezing or breathing with mouth open)
   - Weak breathing (breathing very little)

c. Clonic involuntary movement:
   - None/normal
   - Repetitive mouth/jaw motion
   - Twitching of limb, auricles or head
   - Generalized tremors
   - Clonic seizure
   - Trembling

d. Tonic involuntary movement:
   - None/normal
   - Extending contraction (limb or limbs extends and becomes rigid)
   - Sudden jumping (all limbs come off floor)
   - Tonic extending convulsion
   - Tonic convulsions seen with respiratory distress, seizure or death

e. Vocalization: Check for spontaneous vocalization.

- No vocalization/normal
- Vocalization noted

f. Palpebral closure:
- Open
- Slightly sagging/half shut (eyelids slightly lowered)
- Shut (eyelids are closed)

(2) Hand-held observations

(Observe animals' reactions when they are removed from their cage and held.)

a. Reactivity:
- Very easy (animal sits and allows itself to be picked up)
- Easy/normal (animal does not resist)
- Somewhat difficult (rat stands)
- Difficult (animal cringes and becomes rigid or runs around and is difficult to grab)
- Very difficult (animal attacks)

b. Handling:
- Does not resist/ very easy to handle
- Squeaks or does not squeak but exhibits mild resistance; easy to handle/normal
- Animal freezes and does not move; becomes rigid in hand
- Very difficult (animal struggles, squirms, appears distressed, attempts to bite)

c. Palpebral closure:
- None/normal/ (eyes are open)
- Slightly sagging/half shut (eyelids slightly lowered)
- Shut (eyelids are closed)

d. Lacrimation: Holding the rat, observe areas around eyes.
- No lacrimation/normal
- Moisture only around eyes
- Moisture flows from eyes

e. Salivation: Holding the rat, observe areas around mouth.
- No salivation/normal
- Moisture only around mouth
- Moisture flows from mouth

f. Piloerection: Holding the rat, observe coat (for degree of piloerection).
- None/normal
- Around head and back (mild piloerection only around head and back)
- Generalized (piloerection all over body)

g. Others (to be recorded as description): Note in detail any findings related to dirtiness of hair cast, bite marks, missing nails, gauntness (stomach can be touched, median vertebrae protrude) or death (findings).

(3) Body temperature

A. **During these parameters the animal is still in it`s cage**

1. Body Position:
0 completely flattened
1 lying on a side
2 normal lying (upright)
4 sitting up
6 rearing (on it´s hinterpaws)
8 repeated vertical leaping (jumping)

2. Palpaberal closure:
0 opened
2 ¼ closed
4 ½ closed
6 ¾ closed
8 closed

3. Locomotor activity:
0 none
2 causual scracht, groom/slow. spatial
4 vigor scratch, groom/mod spatial
6 vigor mvmt, rapid
8 extreme mov

4. Bizarre behavior: (-) (none), (+) (Yes)

5. Exopthalmus: (-) (none), (+) (Yes)

6. Respiratory rate:
0 Arrest (none)
2 60/min
4 120/min
6 180/min
8 240+/min

7. Tremors
0 none
2 slightly fine body
4 mod, impaired loc
6 Mod-mkd impair

8. Twitches: 0-8 (frequency)

9. Convulsions: (-) (none), (+) (Yes)

B. **Afterwards the mouse is transfered to a new long cage**

1. Transfer arousal (appearance):
0 coma
1 dulled slow sl mvmt
2 dulled slow mod mvmt
3 Sub alert, sl. dec. mvmt
4 Alert, active mvmt
5 Hiperalert
6 Sl excit, sl sharp
7 Mod escit. Hypomanic
8 Ext. Excit, sharp mvmt manic

2. Spatial locomotion: 0-4 mvmt in 10s multiply by activity: Slow 1, active 1,5, rapid 2

3. Startle response: (chasquear dedos)
0 no reaction
2 ¼ cm
4 ½ cm
6 ¾ cm
8 1+ cm

4. Pilorerection: 0-8 light-extrem

5. Gait: ataxic, hipotonic, impaired (+) or (-)

6. Limb rotation: 0-8 light-extrem

7. Pelvic elevation:
0 flattened
2 barely touches
4 3 mm elevated
6 6mm elevated
8 12 mm elevated

8. Tail elevation:
0 flattened
2 horizontal
4 diagonal 45°
6 vertical 90 °
8 diagonally retrograde 135°

9. Finger approach: *finger 3mm*
0 none
2 head mvment only at distance
4 mvmt to finger no contact
6 contact, partially on finger
8 completely on, explores

10. Finger withdrawal:
0 none
2 eye squint only
4 squint sl head and body retract
6 moderate withdrawal
8 continuous withdrawal

11. Touch escape (under torax press, 3x):
0 none
2 slowly (firm stroke)
4 quick, faster (light stroke)
6 very fast (light stroke)
8 vigorous (barely touch)

12. Positional passivity
(take from the neck) (+) or (-)

13. Visual placing 15 cm high
0 none
1 after nose contact
2 after vibris contact 6mm
3 after sl vibris contact 12mm
4 before vibris contact, active 18mm
5 early vigor extention 15 mm

13. Grip strength
0 none
2 light
4 normal
6 active
8 unusually effective

14. Body tone (between torax and bladder)
0 flacid
2 sl flacid
4 sl resistance
6 mod resistance

15. Pinna (ear stimulation)
0 none
2 sl brisk flick
4 mod brisk flick
6 very brisk flick
8 extrimely hyperactiv

16. Cornea
0 none
2 sluggish closure
4 active single eye blink
6 double eye blink
8 triple eye blink

17. Toe pinch
0 none
2 sl wthdrawal
4 mod rapid withdrawal
6 brisk rapid withdrawal
8 very brisk wthdrawal

18. Wire manouvre
0 actively grasps with HL
2 mod diff grap HL (slowly)
4 mod rapid withdrawal
6 no grasps
8 falls immediately

19. Skin color
0 extr blanching
2 mod blanch, mod pink tone
4 no blanch, sl mod dusky rose
6 deep dusky rose
8 bright deep red flush

20. Diahrrea (+/-)
21. Limb tone 0-8 slight-est resist
22. Provoked biting
0 none
2 slight
4 active no vigorous
6 vigorous, not immediate
8 intensive, cont

23. Tail pinch 4s
0 none
1 slow mvmt, vocal
2 bite, escape
4 mod bite, escape abrupt
6 intensive, cont
8 vigorous bite

24. Righting reflex 2x

Kommunikation

1) **alle -> ¼ jährlich Aufenthaltsraum**
2) **Investigator Team -> monatlich Seminarraum**
3) **Core Team -> wöchentlich Labor**

Telefonnummern

NAME		HANDY	EMAIL	TEL.	PIEPS
Janata	Andreas, Prof.Dr.	0676/88088411	andreas.janata@meduniwien.ac.at	1964	
Schober	Andreas, Dr.	0699/18881972	andreas.schober@meduniwien.ac.at	1956	1950
Sterz	Fritz, Prof.Dr.	0676/4029704	fritz.sterz@meduniwien.ac.at	1952	1952
Warenits	Alexandra, Dr.	0650/3147344	alexandra-maria.warenits@meduniwien.ac.at	1964	1936
Weihs	Wolfgang, Dr.	0660/7376635	wolfgang.weihs@meduniwien.ac.at	5236	
OP				5209	
Labor SA				5236	
Tierpflegez immer				5243	

Dokumentation

Ratte Nr.: Studiengruppe: Markierung: Gewicht- vor Versuch: - post OP:

Versuch Datum:	Datum:<br>24h	Datum:<br>48h	Datum:<br>72h	Datum:<br>Tag 4	Datum:<br>Tag 5	Datum:<br>Tag 6	Datum:<br>Tag 7	Datum:<br>Tag 8	Datum:<br>Tag 9	Datum:<br>Tag 10	Datum:<br>Tag 11	Datum:<br>Tag 12	Datum:<br>Tag 13	Datum:<br>Tag 14
Gewicht														
Körpertemp														
OPC														
NDS														
Consciousness (20)														
Breathing (20)														
Motor (10)														
Sensory (10)														
Corneal reflex (4)														
Olfactory (4)														
Vision (4)														
Wisker Movement (4)														
Hearing (4)														
Travel Ledge (5)														
Placing Test (5)														
Righting Reflex (5)														
Stop at table edge (5)														
Comments														

Versuch Datum:	Datum:<br>24h	Datum:<br>48h	Datum:<br>72h	Datum:<br>Tag 4	
**Visite Vormittag**<br>Uhrzeit<br>Handzeichen Untersuchers					Abbruch
Analgesie					
Flüssigkeitstherapie					
Allg. Pflege					
Kotabsatz					
Harnabsatz					
**Visite Mittag** (falls nötig)<br>Uhrzeit<br>Handzeichen Untersuchers					
Analgesie					
Flüssigkeitstherapie					
Allg. Pflege					
Kotabsatz					
Harnabsatz					
**Visite Nachmittag** (falls nötig)<br>Uhrzeit<br>Handzeichen Untersuchers					
Analgesie					
Flüssigkeitstherapie					
Allg. Pflege					
Kotabsatz					
Harnabsatz					
**Visite Abend** (falls nötig)<br>Uhrzeit<br>Handzeichen Untersuchers					
Analgesie					
Flüssigkeitstherapie					
Allg. Pflege					
Kotabsatz					
Harnabsatz					
Comments					

Wien, am:

für das Exp Team der UKNFM
(A Janata)

für das Exp Team der UKNFM
(W Weihs)

für das Exp Team der UKNFM
(F Sterz)

für das Inst f Biomed Forsch
(H Bergmeister)

für das Inst f Biomed Forsch
(R Plasenzotti)

für das Inst f Biomed Forsch
(N Sagasser)

für das Inst f Biomed Forsch
(S Peiritsch)

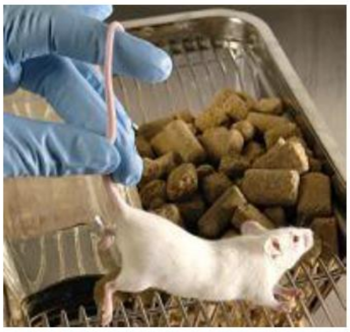

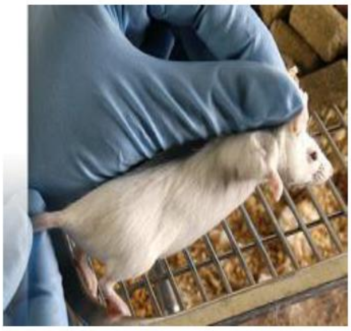

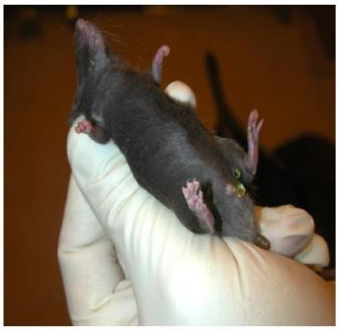

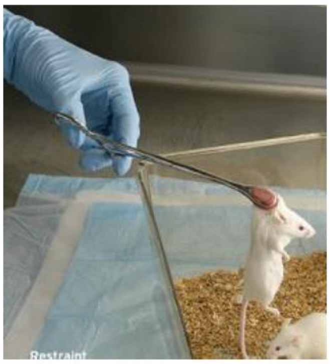

tail restraint

skin fold fixation

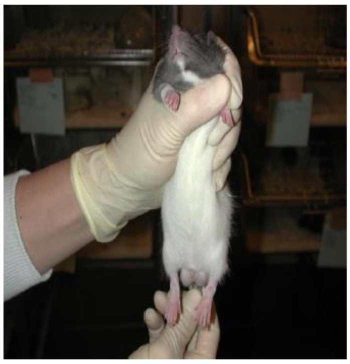

Supplement: S1 Appendix — (PDF) [file pone.0155303.s001.pdf]
