# Supplementary figures and images for: Microdialysis Assessment of Cerebral Perfusion during Cardiac Arrest, Extracorporeal Life Support and Cardiopulmonary Resuscitation in Rats – A Pilot Trial
Source: PLoS One. 2016 May 13;11(5):e0155303. doi: 10.1371/journal.pone.0155303 (PMC4866776; doi:10.1371/journal.pone.0155303)

S1 Fig

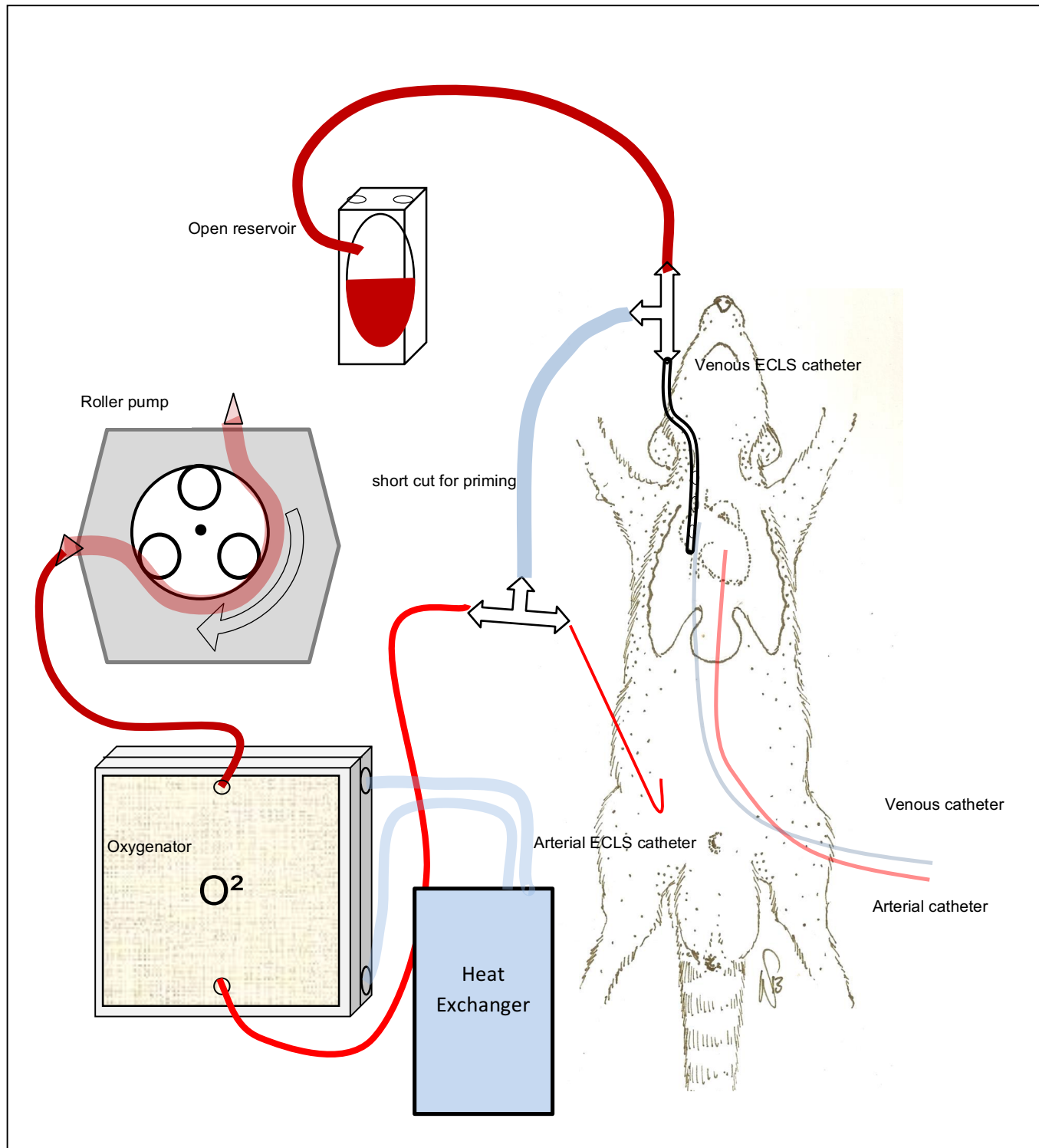

Supplement: S1 Fig — (PDF) [file pone.0155303.s002.pdf]

S2 Fig

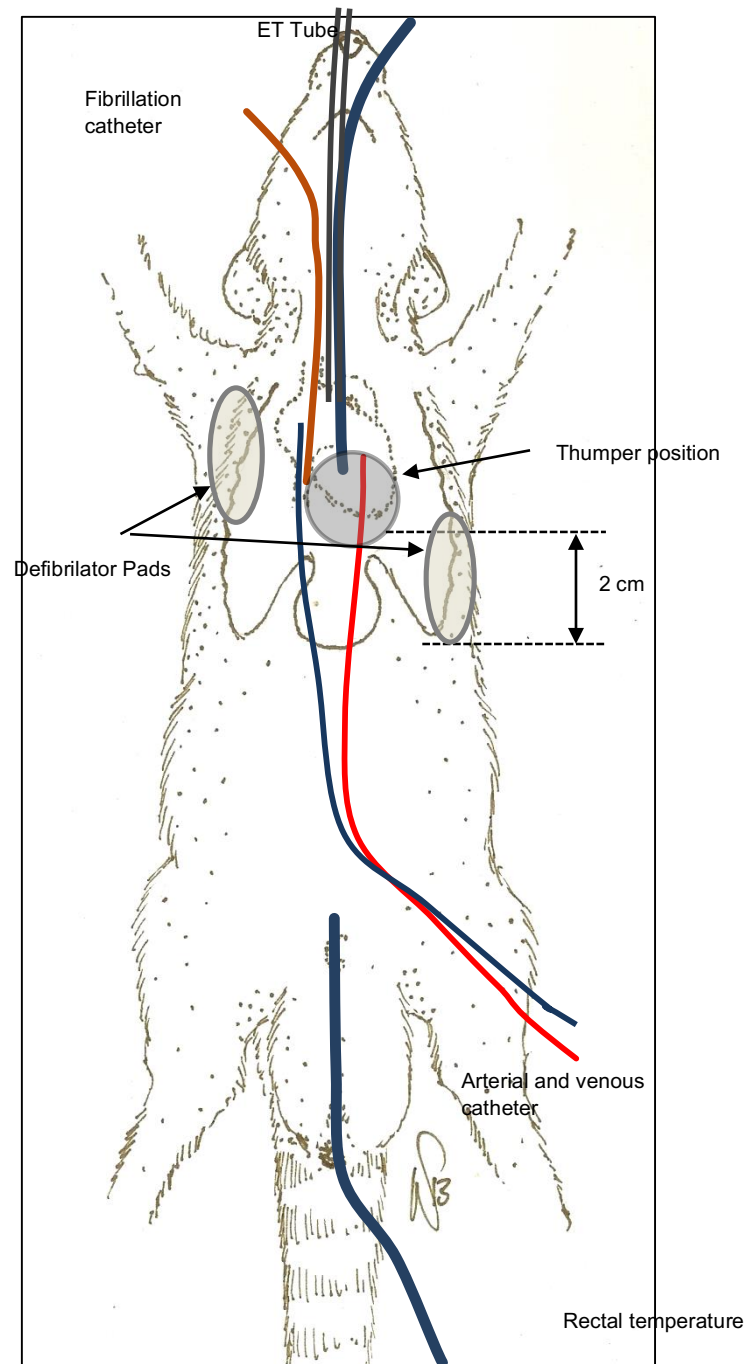

Supplement: S2 Fig — (PDF) [file pone.0155303.s003.pdf]

S3 Fig

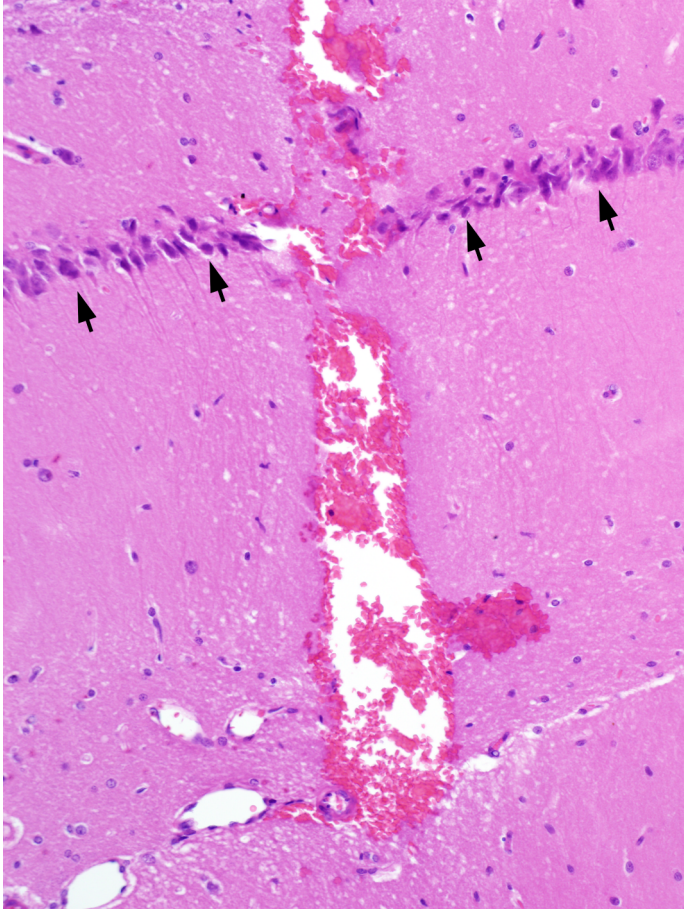

Supplement: S3 Fig — Puncture channel in the hippocampal CA 1 region with mild bleeding; arrows: pyramidal layer of the hippocampus; H&E staining, 200x magnification. (PDF) [file pone.0155303.s004.pdf]

**S4 Fig**

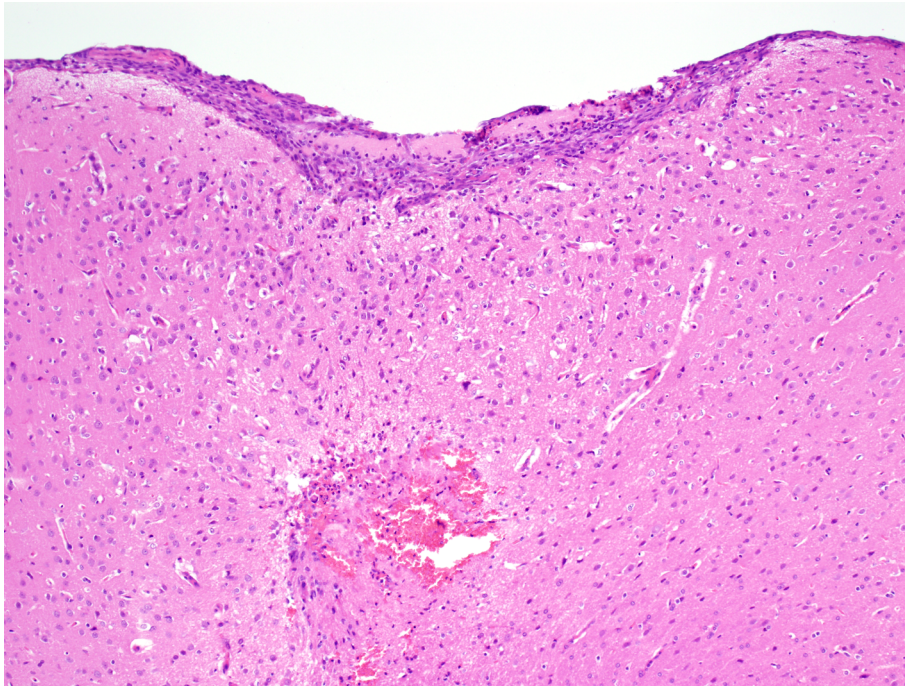

Supplement: S4 Fig — Mild mononuclear infiltration of the meninges, edema and gliosis in the cerebral cortex; H&E staining, 100x magnification. (PDF) [file pone.0155303.s005.pdf]
